# The Role of Career Adaptability and Academic Engagement in College Student’s Life Satisfaction

**DOI:** 10.3390/ijerph21050596

**Published:** 2024-05-05

**Authors:** Íris M. Oliveira, Cátia Marques

**Affiliations:** 1 Faculty of Philosophy and Social Sciences, Centre for Philosophical and Humanistic Studies, Universidade Católica Portuguesa, 4710-362 Braga, Portugal; 2 Research Unit in Education and Community Intervention, Instituto Piaget—ISEIT/Viseu, 3515-776 Viseu, Portugal; catia.marques@ipiaget.pt

**Keywords:** career adaptability, academic engagement, life satisfaction, students, higher education

## Abstract

Career adaptability and academic engagement are important processes in higher education. However, the relationship between these processes and their potential role in students’ life satisfaction still needs to be addressed. The present study aims to explore the role of career adaptability and academic engagement on higher education students’ life satisfaction. This study included 201 participants, 156 women (77.6%) and 45 men (22.4%), aged between 18 and 55 years (*M* = 21.13, *SD* = 4.51). Students answered a sociodemographic questionnaire, the Career Adapt-Abilities Scale, the University Student Engagement Inventory, and the Satisfaction with Life Scale. Positive and statistically significant correlations between career adaptability and academic engagement, as well as between these variables and life satisfaction, were found. The results of a hierarchical linear regression analysis suggested that career adaptability and academic engagement statistically significantly contribute to explaining variations in life satisfaction. This study may lead to a better understanding of the relationship between academic, emotional, and career processes. It may also stimulate integrative psychological practices in higher education settings.

## 1. Introduction

The ongoing social, economic, and political changes have set new challenges in individuals’ career construction [1]. These challenges are also experienced among college students, influencing both their adaptation to and maintenance in higher education, as well as the transition to or increased specialization in the job market. Particularly, both the transitions of entering and leaving higher education are considered important vocational tasks in this stage. These transitions require anticipation and preparation for entering the professional world and performing jobs aligned with training, along with a need to adjust to increasingly complex, changeable, global, and demanding higher education settings [2]. College students need to adapt to changeable academic environments, manage time effectively, and find a balance between studies and personal life [3]. In turn, recent higher education graduates are expected to find or create a job that matches their training and professional goals, make career decisions, and assume new roles in society, often including the professional one, within precarious and unstable labor environments [4,5]. International entities like the Organization for Economic Co-operation and Development (OECD), the United Nations Educational, Scientific, and Cultural Organization (UNESCO), and Eurodyce suggest that the number and diversity of higher education students have increased over the years. The higher education student population currently combines international and national students, mostly emerging and young adults but also middle adults, students who are full-time devoted to academia, and working students who conciliate work and study activities, as well as a representation of various social–economic backgrounds [6,7]. Hence, higher education institutions are challenged to support all students in the development of scientific knowledge and transferable skills [8].

The Life Design paradigm is a theoretical and practical perspective in the career field, which might be useful to help address the current needs of college students and higher education institutions. Based on this paradigm, attention should be given to individuals who are dealing with today’s job market challenges, work-employment processes, and continuous transitions [9]. The main concern of this paradigm is to support individuals’ access to decent jobs and increase their likelihood of experiencing satisfactory life conditions and both personal and professional accomplishments [10]. Career interventions can contribute to addressing these social challenges by promoting a set of psychological resources relevant to the life-designing process, such as career adaptability [5,6].

Career adaptability refers to the psychosocial resources needed to cope with career transitions [1]. It is also conceived as an essential meta-competency in academic/professional environments. As a self-regulation strategy, career adaptability is particularly important in the degree of confidence with which college students face and solve the tasks associated with academic experiences and the transition to the labor market, resulting in a decrease in negative concerns about the future [11]. It is important in higher education, as the way students face and engage with academic demands may, in turn, reflect how they will be in the future and are likely to engage in their roles as workers. Aligned with the importance of career adaptability, academic engagement has also been highlighted in higher education. Academic engagement allows college students to take an active role in the learning processes and take advantage of opportunities, such as new curricular and extracurricular experiences or applying new content and knowledge to practical situations. It is, therefore, an important factor that contributes to the healthy development of individuals [12]. In this way, career adaptability and academic engagement are important processes in higher education, which may hold repercussions on students’ life satisfaction. Students who are more satisfied with their lives, including with their academic life role, seem to be less likely to drop out, present a greater autonomy and ability to persist in academic tasks, self-regulate and properly plan assignments, and engage more in goal-directed behaviors [13]. 

In this scenario, career adaptability and academic engagement emerge as pivotal factors in students’ lives. These factors can directly influence individuals’ life satisfaction and, by extension, their educational success and future professional prospects. Therefore, based on the Life Design paradigm, this study covers the role of career adaptability and academic engagement (as two relevant resources) on higher education students’ life satisfaction. This is an issue of significant social relevance as it may help higher education institutions and policymakers to respond to the needs of students more effectively, thereby contributing to healthier academic environments and, consequently, the advancement of society at large. In addition, this work is aligned with sustainable development goals [14], such as the promotion of Quality Education, Decent Work and Economic Growth, and the Reduction of Inequalities. It relates to the Quality Education goal as it focuses on the life satisfaction of college students and how career adaptability and academic engagement can affect their educational experience and well-being. It also relates to the Decent Work and Economic Growth goal since the concept of career adaptability considers individuals’ abilities to adapt to changes in academic and work environments, which is relevant to promoting decent jobs and sustainable economic growth. Regarding the Reduction of Inequalities, this study can help identify inequalities in students’ life satisfaction, which may provide insights into how to address them. Moreover, if career adaptability or academic engagement impacts students’ life satisfaction, this may highlight inequalities in the previous or current access to opportunities fostering career development and academic engagement. As such, this work can sustain policies and intervention programs for college students aimed at promoting more equal opportunities for academic and career commitment. 

### 1.1. Career Adaptability

The Career Construction Theory (CCT; [15]), aligned with the Life Design paradigm, seeks to explain the interpersonal processes by which individuals organize their personal characteristics, define the direction of their vocational behavior, and assign meaning to their careers [1]. Thus, career is understood as a subjective construction in which the individual plays an active and dynamic role in assigning meanings to past memories, current experiences, and future expectations regarding work and other life roles [16,17]. The CCT presents career adaptability as an important resource for career and life design [18]. Ref. [19] describes a sequence of career adaptation that covers adaptivity readiness (adaptivity), adaptability resources (adaptability), adapting responses (adapting), and adaptation results (adaptation).

Adaptivity readiness includes personality traits and internal variables that configure a disposition to be flexible, proactively face change, and respond to unforeseen events that may arise throughout the career development path, mobilizing a balance between internal needs and the demands of the environment [16,19,20]. For example, adaptivity readiness can be reflected in hopes, desires, and ambitions for the future, which, in turn, are coupled with adaptability resources and adaptive responses, such as engaging in academic and extracurricular tasks or studying in depth. In turn, these interconnections may result in higher satisfaction with the academic role, life, and more favorable academic outcomes. As for the adaptability resources, these shape career adaptability and refer to the psychosocial strengths needed for self-regulation to cope with tasks, transitions, and problems that somehow imply modifications in social integration [21,22]. These resources are described as concern, control, confidence, and curiosity [19]. Concern is the ability to project oneself into the future, considering both what one is and what one would like to become. Control is the predisposition to consider the future as depending on the individual actions and thoughts. Curiosity is the tendency to explore the self, including skills, abilities, knowledge, and values, as well as the environment. Confidence is the belief in one’s ability to face challenges and overcome the barriers that can be experienced in pursuing personal goals [19,23,24]. Additionally, the adapting responses include beliefs and adaptive behaviors that individuals mobilize to cope with career development tasks and changes in academic, work, and living conditions [19,21]. Finally, the adaptation results derive from a process of implementing various resources and responses to articulate internal needs and external opportunities [13]. Examples of adaptation results are one’s perceived success, satisfaction with a decision, career commitment, or life satisfaction [16,20,21]. 

Adaptivity readiness, per se, is not sufficient to support adaptive responses [1]. Self-regulatory and adaptive resources are needed to influence the changing situation. It is through the integration of these elements that adaptive outcomes are achieved [19]. Desired career adaptation outcomes are obtained by individuals who are willing (adaptive readiness) and able (adaptive resources) to perform behaviors that help them cope with changing conditions (adaptive responses) [1,19]. In sum, a successful adaptation is indicated by the individuals’ development and well-being as well as by career satisfaction and success [20,25].

Recent research on career adaptability in higher education underscores the importance of this attribute in the lives of college students. Studies demonstrate that not only perceived academic achievement (e.g., [26]), self-perceived competency, and self-perceived employability (e.g., [27]) but also students’ well-being and life satisfaction (e.g., [28]) are positively linked to career adaptability. The positive relationship between career adaptability and life satisfaction is supported by various components of the career adaptation model, including the ability to establish professional goals, be flexible in the face of uncertainties and changes, and be resilient in pursuing professional goals. These findings underscore the need to actively promote career adaptability as a critical component of educational experiences, with implications for the training of more confident, satisfied, and future-ready students who are about to face upcoming challenges in the job market [5].

### 1.2. Academic Engagement

Higher education provides students with a unique opportunity to develop academic knowledge and a range of career skills. Several career skills are prompted, among other aspects, from the learning processes that occur during the higher education journey. Through academic engagement, students can enhance their critical thinking, problem-solving, and communication and collaboration skills, among others. These skills are not only essential for academic success [29] but also highly valued by employers [30]. 

The way students handle the numerous challenges encountered within their academic setting sustains their ability to manage the professional challenges that lie ahead [31]. Hence, academic engagement is important for students’ achievement, besides following the development of the necessary skills to effectively prepare for the future. Academic engagement is frequently described as the degree of cognitive and behavioral investment students are willing to devote (expectations) or have devoted (behaviors) to higher education life, which, in turn, affects their levels of satisfaction and achievement [32,33]. Academic engagement might be described as a multidimensional concept that includes three dimensions: behavioral, cognitive, and emotional [32,33]. Behavioral Engagement refers to active participation in curricular and extracurricular activities. Cognitive Engagement is related to the use of self-regulatory strategies that facilitate content understanding and learning. Emotional Engagement is reflected in the sense of accomplishment regarding the task, belonging, and academic attachment [33]. A fourth dimension has also been suggested—agentic engagement [34]. This dimension represents the students’ active participation and constructive contribution to creating a more supportive learning environment for themselves [35]. Agentic engagement is related to self-regulated learning as students develop skills to set goals, monitor their own progress, and make informed decisions about their educational paths [36]. Academic environments that foster students’ autonomy, intrinsic motivation, inclusion, and personal agency in their academic choices are, therefore, nurturing satisfaction with the learning experiences, well-being, and preparation for the future [36]. Although most research on academic engagement has focused on middle and high school years, the concept and its underlying principles are applicable and relevant to higher education students. The promotion of higher education students’ autonomy and self-regulation may also enhance the quality of their learning experiences and academic success.

Prior research at the high school level from various continents (e.g., Asia [37,38], North America [39] and Europe [40]) has revealed that students who are more engaged in academic tasks are more satisfied, more committed to the school activities, and more optimistic about the future. High school students who are more academically engaged also show better academic results (e.g., [38,39,40]). Empirical studies [37,38,39] and an integrative review [39] considering higher education students from European, North, and South American contexts have similarly suggested that engagement in academic activities is positively linked to academic achievement and adjustment. On the other hand, students who participate less frequently in class and academic tasks tend to demonstrate a higher risk of dropout [40,41,42]. Thus, academic engagement is an important factor that prevents dropout and academic failure not only in high school [40] but also in higher education [6,42]. Additionally, better academic results and adjustment seem to be associated with greater career adaptability among high school [43] and higher education students [16,42]. Both empirical evidence and integrative reviews suggest that higher education students’ greater career adaptability seems to be linked to higher academic engagement levels [16,41,42]. 

In sum, academic engagement in higher education plays a significant role in the acquisition of knowledge, besides following the development of the career skills necessary to increase the likelihood of future success in the workplace. By investing time and effort into their academic pursuits, college students can develop critical competencies that will serve them well throughout their professional lives.

### 1.3. Life Satisfaction

The study of the impact of decision-making processes and career construction on life satisfaction in college students is justified by the social and organizational transformations that are taking place in the labor market. The Life Design paradigm [24,25] focuses on how individuals construct their lives in the pursuit of personal satisfaction and fulfillment through work. In this way, life satisfaction is an important outcome of career processes [23]. Life satisfaction has been defined in the literature as the cognitive and emotional evaluation that individuals make about different aspects and contexts of their lives [44], and it is often associated with subjective well-being [45,46,47].

In this regard, life satisfaction varies according to the contexts, but also according to each person, it shifts between positive and negative poles [48]. Life satisfaction derives from the interactive and cumulative contribution of different factors [49]. Several studies have related life satisfaction to self-efficacy in adults [50,51] but also to career adaptability among adults [52], adolescents [53], and in specific groups [54]. A literature review consistently found significant relationships between life satisfaction and sociodemographic variables (e.g., age, gender, ethnicity, socioeconomic status), personality, risk behaviors (e.g., drug, alcohol, and tobacco use), and family environment (e.g., parenting styles, family violence) [55]. 

In the higher education context, several studies have explored the relationship between life satisfaction and satisfaction with academic experiences [46,56,57,58], assuming they are important variables in students’ academic adjustment [59,60]. Life satisfaction and satisfaction with academic experiences emerge as preventive factors while facing academic adjustment difficulties [61]. Life satisfaction fluctuates according to the positive or negative way an individual experiences and judges the challenges of the academic context [48]. High levels of life satisfaction have been associated with higher academic engagement and performance, as well as higher levels of self-efficacy and lower academic stress among college students [57]. Academic satisfaction and social support (perceived support, appreciation, and understanding) seem to constitute good predictors of college students’ life satisfaction [45]. Also, psychological resilience in dealing with academic challenges has been associated with life satisfaction [62,63,64,65,66,67]. 

### 1.4. Study’s Aim and Research Hypotheses

Overall, the literature seems to acknowledge the importance of career adaptability, academic engagement, and life satisfaction in higher education students. However, these constructs have often been separately studied, whereby integrative research is still needed. Relying on the Life Design paradigm [9] and the career adaptation model [1,15], this study examines the role of career adaptability and academic engagement in college students’ life satisfaction. Particularly, the following research hypotheses are considered: Hypothesis 1. Career adaptability and academic engagement will be positively related among higher education students; Hypothesis 2. Career adaptability and life satisfaction will be positively related among higher education students; Hypothesis 3. Academic engagement and life satisfaction will be positively related among higher education students; Hypothesis 4. Career adaptability and academic engagement will significantly contribute to explaining variations in life satisfaction among higher education students. This study may help address the need for integrative research identified in the current state of the art. It could also catalyze the development of additional integrative research and psychological practices within higher education settings.

## 2. Materials and Methods

### 2.1. Participants

Participants included 201 college students, 156 females (77.6%), and 45 males (22.4%), aged between 18 and 55 years (*M* = 21.13, *SD* = 4.51; *Mdn* = 20, *IQR* = 1). Following [68], the majority of participants were emerging and young adults (*n* = 196, 97.5%), but there were also five (2.5%) middle adults. Most of the participants were Portuguese (*n* = 198, 98.5%), although there were three (1.5%) participants from Portuguese-speaking countries. Participants were mostly single (*n* = 195, 97%) and devoted full-time to their courses (*n* = 188, 93.5%). However, 13 (6.5%) participants held a working-student status. The majority of participants attended undergraduate studies (*n* = 179, 89.1%), but there were also participants attending Master’s degree studies (*n* = 19, 9.5%) and Higher Technical Professional Courses (*n* = 3, 1.5%). Based on the scientific field categorization adopted by the Portuguese General-Directorate of Higher Education, the fields more frequently represented in the sample included Social and Behavioral Sciences, Entrepreneurial Sciences, Health, and Social Services (65.3%), whereas the less frequently represented fields covered Environment Protection and Security Services (1%). Most of the participants were enrolled in the 2nd or 3rd years of their courses (70.6%). Still, students attending the 1st year (*n* = 32, 15.9%), 4th year (*n* = 15, 7.5%), and 5th year (*n* = 12, 6%) of their courses also participated. Most participants attended Private (*n* = 82, 40.8%) or Public Universities (*n* = 68, 33.8%), with fewer students attending Private (*n* = 11, 5.5%) or Public Polytechnic institutions (*n* = 40, 19.9%).

### 2.2. Measures

Participants answered a social-demographic questionnaire (including questions regarding their sex, age, nationality, marital status, course, attended level of education, grade, and private or public type of higher education institution); the Career Adapt-Abilities Scale (CAAS [19]—Portugal Higher Education Form [69]); the University Student Engagement Inventory (USEI; [70]); and the Satisfaction with Life Scale (SWLS; [44]; adapted by [71,72]). 

The Career Adapt-Abilities Scale (CAAS) consists of 24 items, divided into four subscales of six items each. Each subscale corresponds to a dimension of career adaptability: Concern (e.g., “Thinking about what my future will be like”); Control (e.g., “Keeping upbeat”); Curiosity (e.g., “Investigating options before making a choice”) and Confidence (e.g., “Solving problems”). To answer the items, a five-point Likert-type scale is used, ranging between 1 “not strong” and 5 “strongest”. Higher scores in the total scale and in each subscale are interpreted as higher levels of career adaptability. In the Career Adapt-Abilities Scale—Portugal Higher Education Form [69], Cronbach’s alpha values of 0.92 for the total scale and 0.81, 0.78, 0.84, and 0.86 for the subscales Concern, Control, Curiosity, and Confidence, respectively, were found. 

The University Student Engagement Inventory (USEI; [70]) includes 15 items distributed over three dimensions: Behavioral Engagement (e.g., “I am attentive in class”); Cognitive Engagement (e.g., “I talk to other people outside of school about the subjects I learn in class”); Emotional Engagement (e.g., “I feel excited about schoolwork”). To answer the items, a five-point Likert-type scale is used, ranging between 1 “Never” and 5 “Always”. Cronbach’s alpha values of 0.88 for Emotional Engagement, 0.82 for Cognitive Engagement, and 0.74 for Behavioral Engagement were found [70]. 

The higher education version of the SWLS (SWLS; [44,71]) for Portuguese students is made up of five items intended to evaluate the global cognitive judgments of an individual’s satisfaction with life (e.g., “In most ways my life is close to my ideal”). Items are answered on a seven-point Likert-type scale, ranging from 1 “Totally disagree” to 7 “Totally agree”. Higher scores are interpreted as greater life satisfaction. In the original version [44], a Cronbach’s alpha coefficient of 0.82 was obtained. With Portuguese higher education students, [46] found a Cronbach’s alpha of 0.77. The internal consistency index obtained was close to the one found in other studies with Portuguese samples (e.g., [71,72]). 

### 2.3. Procedures

Data were collected in an online format in the middle of the second semester in Portuguese higher education institutions, with invitations for participation presented to the higher education students’ community through class contacts, email, and social media. Students were asked to complete the questionnaires and share with other colleagues who wished and could be available to participate in the study. A non-probabilistic snowball sampling approach was, therefore, used. Given the increased heterogeneity of the higher education student population, participation in this study was afforded to individuals of 18 years or older, who either studied full-time or were working students, who were Portuguese or of other nationality (if they understood the Portuguese oral/written language), and who attended undergraduate or graduate courses in public or private institutions. Participants were informed about the purpose of the study and were also assured that all information was anonymous, treated confidentially, and used exclusively for research purposes. Only after consenting to their participation did students have access and complete the online protocol. Additional ethical research considerations were assured, relying on the Code of Ethics from the Portuguese Order of Psychologists [73] and on the APA Ethical Principles of Psychologists and Code of Conduct [74].

### 2.4. Analyses

The Statistical Package for the Social Sciences (SPSS), version 28 for Windows, was used for data analysis. Preliminary analyses associating participants’ academic years (i.e., 1st, 2nd, 3rd, 4th, and 5th) and contexts (i.e., public or private universities, public or private polytechnic institutes) with career adaptability, academic engagement, and life satisfaction were performed. Given the scale of measurement of the variables, Spearman correlation coefficients were computed to explore associations among the academic years and the constructs. Chi-square tests were used to investigate associations with the academic contexts. Descriptive statistics was used to describe the variables in the sample, as well as parametric (i.e., Pearson) and non-parametric (i.e., Spearman) correlation coefficients were computed to test correlations between career adaptability, academic engagement, and life satisfaction due to evidence of univariate non-normality attested by the Kolmogorov–Smirnov test. When both correlation coefficients offered consistent results regarding the rejection or retention of the null hypothesis, parametric results were reported [75].

Subsequently, hierarchical linear regression analysis was performed to test the effect of career adaptability and academic engagement on life satisfaction. Four assumptions for conducting the regression analysis were previously verified [76]. The assumption of independence of observations was ensured based on a Durbin–Watson value of 1.94. The absence of singularity and multicollinearity assumption was attained by finding correlations lower than 0.90, a Variance Inflation Factor (VIF) value of 1.40, and a Tolerance value of 0.7. As for the assumption regarding the absence of outliers, the Cook’s Distance statistic was lower than 1 (Cook’s D = 0.109), and the standardized residuals fell within the range of −3 to 3 (respectively = −2.67 and 2.41), thus ensuring the absence of any influential or extreme data points. The assumption of normality of the distribution of standardized residuals was also assessed and guaranteed (ZRE Kolmogorov–Smirnov *p* = 0.20). These collective assumptions evaluations fortified the possibility of conducting the regression analysis. The hierarchical linear regression analysis was carried out, entering career adaptability in the first step/block and academic engagement in the second step/block of the explaining variables and assuming life satisfaction as the outcome variable.

## 3. Results

Preliminary results suggested that the academic years were positively and statistically significantly associated with career adaptability (*r_s_* = 0.21, *p* = 0.003), academic engagement (*r_s_* = 0.17, *p* = 0.02), and life satisfaction (*r_s_* = 0.18, *p* = 0.01). Students who were more advanced in their higher education path presented greater levels of career adaptability, academic engagement, and life satisfaction. No associations between the academic contexts and the constructs were found.

Participants registered minimum and maximum scores compatible with the measure’s possible scores. Students showed lower average levels of curiosity compared to the other career adaptability dimensions. Regarding academic engagement, students showed medium levels (54.45), higher than the possible mean point of the scale (36.5). The average score of life satisfaction registered by the students (17.37) was higher than the possible mean point of the scale (12.5), which indicates positive values of life satisfaction. 

Correlation results are presented in Table 1. The intra-scale correlation results suggest that there are positive and statistically significant correlations between the dimensions of career adaptability (e.g., *r* = 0.62, *p* < 0.001—students with greater levels of confidence present greater levels of control). All the career adaptability dimensions and the CAAS total score are positively and statistically significantly related (concern *r* = 0.26, *p* <.001; curiosity *r* = 0.37, *p* < 0.001; control *r* = 0.48, *p* < 0.001; confidence *r* = 0.40, *p* < 0.001). Similarly, all the dimensions of academic engagement are positively and statistically significantly correlated with each other (e.g., *r* = 0.55, *p* < 0.001—students with greater Cognitive Engagement present greater Behavioral Engagement). Positive and statistically significant correlations between all the academic engagement dimensions and the total score of the scale were also found (Cognitive Engagement *r* = 0.84, *p* < 0.001; Behavioral Engagement *r* = 0.83, *p* < 0.001; Emotional Engagement *r* = 0.83, *p* < 0.001).

The inter-scale correlation results show that there are positive and statistically significant correlations between career adaptability and academic engagement. Higher levels of concern (*r* = 0.31, *p* <.001), control (*r* = 0.44, *p* < 0.001), curiosity (*r* = 0.48, *p* < 0.001), and confidence (*r* = 0.51, *p* < 0.001) are statistically significantly associated with higher levels of academic engagement. Moreover, all career adaptability dimensions are positively related to all academic engagement dimensions. The total career adaptability score is also positively related to the academic engagement dimensions and total score. 

The inter-scale correlation results also show a positive and statistically significant correlation between career adaptability and life satisfaction (*r* = 0.46, *p* < 0.001). In this way, greater career adaptability levels, and specifically greater concern, control, curiosity, and confidence levels, are statistically significantly associated with higher life satisfaction. 

All the academic engagement dimensions (Cognitive Engagement *r* = 0.38, *p* < 0.001; Behavioral Engagement *r* = 0.38, *p* < 0.001; Emotional Engagement *r* = 0.54, *p* < 0.001), as well as its total score (*r* = 0.53, *p* < 0.001) are positively related with life satisfaction. Higher academic engagement is statistically significantly associated with higher life satisfaction.

As for the results from the hierarchical linear regression model, the first block explains 51% of the variance (Adjusted *R*^2^ = 0.26) and is statistically significant, *F* (1, 199) = 69.33, *p* < 0.001. Career adaptability constitutes a statistically significant variable explaining life satisfaction (β = 0.54, *t* = 8.33, *p* < 0.001). Adding the second regression block, the model explains 59% of the variance (Adjusted *R*^2^ = 0.35) and is statistically significant, *F* (1, 198) = 53.05, *p* < 0.001. Career adaptability remains a statistically significant variable explaining life satisfaction (β = 0.32, *t* = 4.68, *p* < 0.001), as well as academic engagement (β = 0.36, *t* = 5.25, *p* < 0.001). In this way, career adaptability and academic engagement are identified as statistically significant variables explaining life satisfaction. Greater career adaptability and greater academic engagement are associated with higher life satisfaction (Table 2).

## 4. Discussion

This study sought to examine the role of career adaptability and academic engagement in college students’ life satisfaction. The Life Design paradigm [9] and the career adaptation model [1,15] served as the theoretical lenses for this study. Based on such a theoretical background, this study assumed the importance of career adaptability as a key meta-competency to navigate transitions and achieve satisfactory life conditions, as well as of academic engagement as a related pivotal process in higher education. The Life Design paradigm [9] and the career adaptation model [1,15] can help address the current needs of higher education students and institutions, as the main concern of this theoretical framework is to support individuals’ access to decent jobs and adaption to various challenges and transitions, keeping one’s integrity and orientation to personally meaningful goals while navigating unpredictable and precarious environments [9,19]. Relying on this framework, career interventions can support individuals’ flourishing and holistic development through an inter-related view of human processes, as well as contribute to individuals’ psychosocial adjustment and well-being through the promotion of a set of psychological resources relevant to the life-designing and career adaptation process, such as career adaptability [9,18]. This study investigated the joint contribution of career adaptability and academic engagement to higher education students’ life satisfaction, thus offering an integrative input that is still required in this line of research and holds the potential to inform practices. 

Preliminary results indicated positive associations between students’ academic years and career adaptability, academic engagement, and life satisfaction. This may suggest that as students advance in their academic journey, they improve the skills required to adapt to academic and career challenges, become more committed to their academic pursuits, and experience increased life satisfaction. It might be the case that as students advance in their higher education path, they benefit from the accumulation of a set of experiences that help them develop personal resources and feel more confident in their abilities to adaptively deal with academic and career changes. As students approach the final years of their courses, they may also be more open to exploring possibilities to keep pursuing studies leading to higher academic degrees and/or applying to professional positions aligned with their training [5]. This more immediate concern with academic/career transitions coupled with the openness to explore potential avenues to pursue after completing a course might prompt students’ activation of career adaptability resources, increased engagement in academic endeavors, and a sense of accomplishment and satisfaction. Nonetheless, these preliminary findings may also be illustrative of the fact that some students attending the first years of college struggle to understand how an academic course and both academic and extracurricular activities are important for and linked to their personal development and career readiness [6]. Hence, these preliminary findings might point to the need to implement preventive and promotional practices during the higher education journey to better help students integrate their academic experiences with their career goals, as well as to systematically foster their development of transferrable skills and well-being. To do so, mentoring activities, student-centered pedagogical practices, career self-management programs, and infusion of career experiences into academic programs could be useful [5,6,7].

Additionally, preliminary results did not find significant associations between the academic contexts (i.e., public or private universities, public or private polytechnic institutes) and the covered constructs. These findings may suggest that both public and private universities and higher education institutes are similarly committed to the promotion of students’ scientific knowledge and transferrable skills in alignment with higher education policies and worldwide discussions [6,7,8]. Still, research relying on the Life Design paradigm [9] and on the career adaptation model [1,15] has mostly considered personal influences on the adaptation process. Studies covering contextual influences and interactions between personal and contextual factors in individuals’ career adaptation are, therefore, still needed in this line of inquiry [5].

However, it is important to emphasize that the associations between students’ academic years and contexts with career adaptability, academic engagement, and life satisfaction were not the focus of this study but rather served as preliminary information. Hence, it would be important to conduct additional cross-sectional and longitudinal studies to deepen knowledge regarding variations in these core constructs for academic years and academic contextual features, such as the type of higher education institution, its geographical location, or its learning climate. These research efforts could ultimately hold implications for educators, policymakers, and career counselors. Understanding how career adaptability, academic engagement, and life satisfaction vary throughout the higher education journey and according to personal and contextual factors would be useful to build interventions that better respond to the needs of a diverse college student population, preparing them to adaptively cope with academic challenges and career transitions [5,27]. 

This study offered evidence to support Hypothesis 1, as a positive association between career adaptability and academic engagement was found. Academic engagement can serve as a foundation for developing the skills and knowledge necessary for future career changes. Students who perceive their higher education experiences as a part of a continuous process of skill, personal, and professional development may approach their studies with greater enthusiasm and commitment, thus taking advantage of opportunities that may be useful for their overall development and career readiness [6,16]. Additionally, students who are curious and concerned about the future, take control over their actions, and are confident about their potential are likely to respond better to higher education challenges. Hence, these results are aligned with literature suggesting that students who are more engaged in and proactively cope with academic tasks are more committed to the course and more optimistic about the future [13,27,33]. Individuals equipped with the psychosocial resources of career adaptability seem to be better positioned to actively participate in their academic experiences. Adaptable individuals seem to be more prepared to navigate academic challenges, maintaining a focused and proactive disposition toward the ongoing construction of their career and life projects. However, these results also call attention to students presenting low career adaptability levels, as they seem to simultaneously be weakly engaged in academic activities. Two main implications for practice can be discussed based on these results. On the one hand, it is crucial to invest in interventions that promote students’ career adaptability throughout the mandatory school years. Although most career interventions in the mandatory school path tend to occur when students are approaching expected career decision moments, more systematic work to foster career adaptability resources throughout the lifespan is needed [19,53]. Direct career interventions with students and indirect interventions embracing collaboration with teachers, parents, school leaders, and the community can be implemented to offer pupils opportunities to explore various jobs, career paths, and lifestyles, to raise self-knowledge regarding one’s interests, values, and strengths, as well as to help them understand the role of school and academic experiences for the future. Such systematic work might even support more informed and confident decisions regarding enrolment in higher education. On the other hand, higher education institutions should invest in programs (e.g., mentoring, volunteer work) and services (e.g., psychology, career counseling) aimed at supporting students’ adaptation to college, development of transferable skills (including career adaptability) throughout the academic journey, and transition to the working world and employment [2,5,13,16].

The results from this study also supported Hypothesis 2 since a positive association between career adaptability and life satisfaction was found. These findings are consistent with literature indicating that career adaptability is an important resource for individuals’ well-being [20,62]. Individuals demonstrating higher levels of career adaptability are more likely to experience greater satisfaction with their lives. Students who engage in the exploration of themselves and their surroundings and who feel confident in making informed decisions about their education and future careers are likely to experience higher levels of life satisfaction. The role of career adaptability in higher education is, therefore, emphasized in this study. Career adaptability competencies can be transferrable to academic settings, leveraging students with greater autonomy and ability to persist in academic tasks, self-regulate, properly plan academic assignments, engage in goal-directed behaviors, and be academically satisfied [11,13,16,27]. Based on the Life Design paradigm [9] and on the career adaptation model [1,15], career counseling services in higher education institutions could offer valuable contributions to promote students’ career adaptability and well-being through collaborative practices with the academic community [5]. These services could also implement individual career counseling sessions to help students assign meaning to their previous and current experiences and establish personally meaningful goals and life projects, thus prompting their adaptation to challenges and life satisfaction [5,24,25].

Hypothesis 3 was also supported in this study, as a positive association between academic engagement and life satisfaction was verified. Academic engagement can be linked to a sense of purpose and meaning in students’ lives, whereby when students find value and relevance in their academic pursuits, they can obtain a sense of direction, accomplishment, and satisfaction [47,57]. These results seem to support prior research indicating that students who are more engaged in their academic responsibilities are also more satisfied, more committed to the course, and more confident about the future [6,13,31]. Hence, active teaching–learning practices, including the development of projects, the organization of visits to community institutions, or the use of technology to create innovative learning assets/outputs [7], could be considered to foster higher education students’ academic engagement and life satisfaction. Teachers’ attention to the needs of their students, modeling of respectful and empathic relations, autonomy-supportive practices, and combination of academic content with students’ career goals, suggestions, experiences, and curiosity might contribute to their motivation, engagement, and satisfaction [35,37,42].

The results also supported Hypothesis 4, as career adaptability and academic engagement were shown to significantly contribute to explaining variations in life satisfaction. Aligned with other studies that separately covered these constructs (e.g., [18,20,57]), career adaptability seems to be positively linked to academic engagement. Moreover, career adaptability and academic engagement, both separately and jointly, seem to impact college students’ life satisfaction. The interconnection among these constructs is a crucial finding, as life satisfaction is not only a valuable outcome but also an indicator of overall well-being and resilience. Investing in the promotion of career adaptability seems crucial to assist individuals in adapting and thriving in changeable academic and work environments, fostering commitment and development, and thus contributing to the well-being and success of both higher education students and organizations. These results support a holistic and integrative view of individuals, acknowledging their several life arenas and agencies dealing with changes while remaining loyal to their career goals and building personally meaningful projects [24,47]. Hence, higher education institutions need to respect the person of each student and bring the academic community together to offer multiple services and opportunities inside and outside the classroom that better respond to the student’s needs [5,7,48,57]. In doing so, higher education institutions might afford students the opportunity to become better equipped to face the challenges of contemporary society and attain their full potential and well-being.

Despite the contribution of this study, it is important to acknowledge some limitations and implications for future research. First, this study employed a non-probabilistic sampling method, which calls for caution in the generalization of the results. Second, the gender composition of the sample was unbalanced, with a higher frequency of women than men. Although statistical records shared by entities like the OECD and UNESCO suggest a worldwide trend of more women attending higher education than men, it would be useful to capture a more balanced gender representation in future studies. Third, there was an unbalanced distribution of participants per academic year and context, which would be important to overcome in future research. Hence, this study could be replicated with a larger, more representative, and balanced sample of higher education students, following a probabilistic stratified random sampling method to overcome these limitations. Fourth, this study enlightened the possibility of conducting integrative research on the career adaptability, academic engagement, and life satisfaction constructs in higher education, which have so far been mostly separately covered. However, it presented a cross-sectional nature, which does not enable addressing these constructs’ interconnections over time. Even though these results highlight the relationship between these constructs, future longitudinal studies might be useful to better understand the dynamics of these relationships throughout students’ higher education path. Fifth, although agentic engagement has been acknowledged as a fourth dimension of students’ academic engagement [34], the measure herein used did not enable the assessment of such a dimension. Although the selected measure presents documented favorable psychometric qualities [33], it would be useful to add an assessment of students’ agentic engagement to attain a more complete understanding of the phenomena in higher education. 

## 5. Conclusions

Based on this study, several possibilities for future research can additionally be considered. Based on the Life Design paradigm [9] and the career construction model [1,15], future research could add other variables indicative of one’s adaptivity/adaptive readiness to the model (e.g., personality), thus conducting a complete test of the career adaptation model targeting higher education students’ life satisfaction. It could also be useful for future studies to add evidence on students’ academic achievement as another adaptation result. Through correlational and/or structural equation analyses, such studies could explore relations between the constructs herein covered with academic achievement while contributing to a more holistic understanding of students’ career adaptation and academic success [27,43,48]. Moreover, as the promotion of students’ transferrable skills and well-being is a global concern among higher education policymakers and institutions [7,8], future cross-cultural studies could be performed. These studies could help identify commonalities and differences in higher education students’ career adaptability, academic engagement, and life satisfaction across countries, thus fostering international research collaboration on the topic. Future research could also explore variations in these constructs for students’ developmental stages. Although the higher education student population mostly includes emerging and young adults, middle adults have also been enrolling in college [6,7]. As emerging, young, and middle adulthood feature different developmental challenges [68], it would be useful to conduct comparative quantitative studies, mixed-method, or case studies to better capture the specific career and academic needs of higher education students according to their developmental stage. 

Practical implications from this study can also be generally considered. Higher education institutions, policymakers, and practitioners can use this study’s evidence to sustain the development of integrative interventions aimed at enhancing career adaptability, academic engagement, and life satisfaction among students. Active teaching practices, opportunities for extracurricular activities, and career counseling services are important to respond to college students’ needs and to promote their overall development and life satisfaction. By fostering these psychological resources, institutions can contribute not only to students’ academic success but also to transferable skills needed to embrace future challenges, life satisfaction, and the attainment of sustainable development goals [6,13,14].

In conclusion, this study offers valuable input to bridging the gap from fragmented research to more integrative research examining career adaptability, academic engagement, and life satisfaction among higher education students. By emphasizing the interconnectedness among these constructs, this study might open potential avenues to advance research on the topic and to sustain interventions and policies that nurture healthier academic environments and students’ well-being. Overall, higher education institutions are no longer solely focused on the construction and transmission of scientific knowledge; they instead play a critical role in nurturing individuals’ holistic development and agency, as well as contributing to positive and transformative learning environments that support the flourishing of each individual and society. 

## Figures and Tables

**Table 1:** Descriptive and Correlation Results.

	Min	Max	*M*	*SD*	1.	2.	3.	4.	5.	6.	7.	8.	9.
1. Concern	11	30	23.51	3.29									
2. Control	14	30	23.80	3.30	0.42 ***								
3. Curiosity	11	30	22.64	3.43	0.47 ***	0.58 ***							
4. Confidence	8	30	23.55	3.40	0.45 ***	0.62 ***	0.61 ***						
5. CA Total	51	118	93.50	10.97	0.71 ***	0.82 ***	0.83 ***	0.81 ***					
6. CE	7	25	18.34	3.22	0.26 ***	0.32 ***	0.47 ***	0.41 ***	0.44 ***				
7. BE	10	25	19.00	2.64	0.30 ***	0.38 **	0.38 ***	0.40 ***	0.46 ***	0.55 ***			
8. EE	5	25	17.12	3.43	0.10 ***	0.33 ***	0.31 ***	0.29 ***	0.32 ***	0.42 ***	0.42 ***		
9. Engag. Tot	22	73	54.45	7.75	0.31 ***	0.44 ***	0.48 ***	0.51 ***	0.54 ***	0.84 ***	0.83 ***	0.83 ***	
10. L. Satisf.	5	25	17.37	3.74	0.26 ***	0.48 ***	0.37 ***	0.40 ***	0.46 ***	0.38 ***	0.38 ***	0.54 ***	0.53 ***

Note: ** *p* < 0.01; *** *p* < 0.001. CA Total = Career Adaptability; CE = Cognitive Engagement; BE = Behavioral Engagement; EE = Emotional Engagement; Engag. Tot. = Engagement Total; L. Satisf. = Life Satisfaction.

**Table 2:** Hierarchical Linear Regression Results.

	*R*^2^ (*R*^2^Adj.)	Model	β	*t*
First Block Career Adaptability	0.26 (0.26)	*F* (1, 199) = 69.33 ***	0.54	8.33 ***
Second Block Career Adaptability and Engagement	0.35 (0.34)	*F* (2, 198) = 53.05 ***	0.32 0.36	4.68 *** 5.25 ***

*** *p* < 0.001.

## Data Availability

The data supporting the findings of this study are available from the authors, upon reasonable request.
